# Cross-species transcriptomics identifies core regulatory changes differentiating the asymptomatic asexual and virulent sexual life cycles of grass-symbiotic *Epichloë* fungi

**DOI:** 10.1093/g3journal/jkac043

**Published:** 2022-02-22

**Authors:** Daniel Berry, Kate Lee, David Winter, Wade Mace, Yvonne Becker, Padmaja Nagabhyru, Artemis D Treindl, Esteban Valverde Bogantes, Carolyn A Young, Adrian Leuchtmann, Linda J Johnson, Richard D Johnson, Murray P Cox, Christopher L Schardl, Barry Scott

**Affiliations:** Institute of Fundamental Sciences, Massey University, Palmerston North 4442, New Zealand; Institute of Fundamental Sciences, Massey University, Palmerston North 4442, New Zealand; Institute of Fundamental Sciences, Massey University, Palmerston North 4442, New Zealand; AgResearch Ltd, Grasslands Research Centre, Palmerston North 4442, New Zealand; Institute for Epidemiology and Pathogen Diagnostics, Julius Kühn Institute, Federal Research Centre for Cultivated Plants, 38104 Braunschweig, Germany; Department of Plant Pathology, University of Kentucky, Lexington, KY 40546, USA; Institute of Integrative Biology, ETH Zurich, 8092 Zürich, Switzerland; Department of Plant Biology, Michigan State University, East Lansing, MI 48823, USA; Noble Research Institute, LLC, Ardmore, OK 73401, USA; Institute of Integrative Biology, ETH Zurich, 8092 Zürich, Switzerland; AgResearch Limited, Palmerston North 4442, New Zealand; AgResearch Limited, Palmerston North 4442, New Zealand; Institute of Fundamental Sciences, Massey University, Palmerston North 4442, New Zealand; Department of Plant Pathology, University of Kentucky, Lexington, KY 40546, USA; Institute of Fundamental Sciences, Massey University, Palmerston North 4442, New Zealand

**Keywords:** Epichloë, stroma, mutalism, virulence, plant-fungal interactions, RNAseq

## Abstract

Fungi from the genus *Epichloë* form systemic endobiotic infections of cool season grasses, producing a range of host-protective natural products in return for access to nutrients. These infections are asymptomatic during vegetative host growth, with associations between asexual *Epichloë* spp. and their hosts considered mutualistic. However, the sexual cycle of *Epichloë* spp. involves virulent growth, characterized by the envelopment and sterilization of a developing host inflorescence by a dense sheath of mycelia known as a stroma. Microscopic analysis of stromata revealed a dramatic increase in hyphal propagation and host degradation compared with asymptomatic tissues. RNAseq was used to identify differentially expressed genes in asymptomatic vs stromatized tissues from 3 diverse *Epichloë*–host associations. Comparative analysis identified a core set of 135 differentially expressed genes that exhibited conserved transcriptional changes across all 3 associations. The core differentially expressed genes more strongly expressed during virulent growth encode proteins associated with host suppression, digestion, adaptation to the external environment, a biosynthetic gene cluster, and 5 transcription factors that may regulate *Epichloë* stroma formation. An additional 5 transcription factor encoding differentially expressed genes were suppressed during virulent growth, suggesting they regulate mutualistic processes. Expression of biosynthetic gene clusters for natural products that suppress herbivory was universally suppressed during virulent growth, and additional biosynthetic gene clusters that may encode production of novel host-protective natural products were identified. A comparative analysis of 26 *Epichloë* genomes found a general decrease in core differentially expressed gene conservation among asexual species, and a specific decrease in conservation for the biosynthetic gene cluster expressed during virulent growth and an unusual uncharacterized gene.

## Introduction

*Epichloë* spp. are fungal symbionts of cool season grasses that can exhibit both asymptomatic asexual and virulent sexual life cycles, depending on the host and *Epichloë* species involved (Schardl 2010). Growth in host vegetative tissues is strictly regulated, establishing a sparse yet systemic network of endobiotic hyphae throughout the intercellular spaces of host aerial tissues, with the exception that host vascular bundles are usually not colonized (Tanaka *et al.* 2006; Christensen *et al.* 2008). These endobiotic hyphae are also able to form plant exit structures, called expressoria, enabling colonization of leaf surfaces with a similarly sparse network of epibiotic hyphae (Becker *et al.* 2016). Infected host plants are visually asymptomatic, and typically benefit from herbivore-suppressing natural products produced by the fungal symbiont (Schardl 2010; Schardl *et al.* 2013), with infected plants often outperforming their uninfected counterparts (Johnson *et al.* 2013a). The asexual life cycle of *Epichloë* fungi is thus characterized as mutualistic; however, the sexual cycle has a detrimental impact on the grass host (Chung and Schardl 1997). This is because the fungal sexual phase starts with the formation of a presexual structure, known as a stroma (plural: stromata), which consists of a dense sheath of hyphae that encases and aborts the developing grass inflorescence, thus impeding the plant’s sexual cycle in favor of its own (Schardl *et al.* 2004). The dichotomy between the asymptomatic asexual and virulent sexual life cycles of *Epichloë* spp. presents an opportunity to investigate the mechanisms differentiating plant-mutualists from plant-pathogens within a single genetic system.

Stroma formation starts with the hyper-proliferation of epibiotic hyphae to form dense layers of fungal hyphae that separate and surround host inflorescence tissues, suppressing flowering (Schardl and Leuchtmann 2005). The endobiotic hyphae contained within these enveloped plant tissues also undergo hyper-proliferation, and extensively colonize host vascular bundles, presumably facilitating expropriation of host nutrients to fuel the fungal sexual cycle. Immature stromata form conidia on their surface that act as spermatia, and produce volatile compounds to attract flies from the genus *Botanophila* that act as fertilization vectors for gamete dispersal (Schiestl *et al.* 2006; Bultman and Leuchtmann 2008; Steinebrunner *et al.* 2008). These flies consume external stromal tissues, and fertilization occurs when ingested conidia are deposited through defecation on a stroma of the opposite mating type (Bultman *et al.* 1995, 1998). Fertilized stromata change color from white to orange-yellow and form meiotically derived ascospores within perithecia located just below the stroma surface. Once expelled, these ascospores mediate contagious horizontal transmission to uninfected host plants. *Botanophila* flies also lay their eggs on stromata and their larvae subsist upon perithecial tissues until pupation; the flies, therefore, benefit from the fungal sexual cycle. Importantly, stromata have well-defined boundaries that do not spread beyond the immediate proximity of the emerging host inflorescence, with the stem of the host reproductive tiller located immediately below each stroma remaining visually asymptomatic (Bultman and Leuchtmann 2008).

Although stroma formation is conserved across all sexual *Epichloë* species, the frequency of stroma formation varies between different *Epichloë* species and strains (Schardl and Leuchtmann 2005). Sexual species may consistently form stromata and thereby strongly suppress host plant seed formation, or they may form stromata at an intermediate frequency such that plants exhibit a mix of symptomatic and asymptomatic inflorescences. Hyphae in asymptomatic inflorescences can disseminate to a subsequent generation of plants through infection of the host seed, and asexual *Epichloë* species are exclusively transmitted via this vertical mechanism (Schardl and Leuchtmann 2005). Therefore, while asexual *Epichloë* species can be considered true mutualists, sexual *Epichloë* species exist on a spectrum that ranges from predominantly mutualistic through to pathogenic depending on the frequency of stroma formation. The signaling pathways that control the transition from asymptomatic growth in host vegetative tissues to the virulent growth associated with stroma formation are not known, nor are the mechanisms involved in stroma formation well understood. Here, we take advantage of the conserved nature of stroma formation within the *Epichloë* genus to explore the gene expression changes specifically associated with the switch from asymptomatic to virulent growth during stroma formation. This is achieved by comparing 3 diverse *Epichloë–*host associations. These include the moderately antagonistic *Epichloë elymi*/*Elymus virginicus* and *Epichloë festucae*/*Schedonorus pratensis* associations, which form stromata at intermediate frequencies, and the highly antagonistic *Epichloë typhina/Lolium perenne* association, which forms stromata on every infected reproductive tiller.

## Materials and methods

### RNA sample preparation and sequencing

Established *S.* *pratensis*, *E.* *virginicus*, and *L.* *perenne* plants infected with *E.* *festucae* strain E2368, *E.* *elymi* strain NFe728, and *E.* *typhina* strain E8, respectively, were vernalized under cover outdoors in Lexington, Kentucky over the 2015–2016 northern hemisphere winter. Whole emerging stromata (STR) were sampled from each association as they presented in spring 2016, as were substromal samples (SUB), consisting of a reproductive stem section taken from 1 to 5 cm below the base of each STR sample. Emerging asymptomatic infected inflorescence (INF) samples were also harvested from the *E. elymi* and *E. festucae* associations; INF samples were unavailable for the *E. typhina* association due to the obligate stroma-forming nature of this *Epichloë* isolate. All STR, SUB, and INF samples were harvested from a single plant for each association. Samples were snap-frozen in liquid nitrogen, stored at −80°C, then extracted for RNA using the Qiagen RNeasy Plant Mini Kit (Qiagen, Venlo, The Netherlands) as per the manufacturer’s instructions. Three independent RNA samples for each tissue type from each association were selected for sequencing following RNA integrity testing by Experion (Bio-Rad, Hercules, CA, USA), with all selected samples exhibiting RNA quality indicator (RQI) values above 8.8. Wherever possible, STR and SUB samples sharing the same numerical suffix (e.g. Eel-STR1 and Eel-SUB1) were sourced from the same reproductive tiller, with the sole exception that Eel-STR3 and Eel-SUB3 had to be sourced from different tillers to meet RQI requirements. Previous studies showed that *Epichloë*-derived reads typically account for only 2–8% of the total mRNA pool from infected host vegetative tissues (Dupont *et al.* 2015), and we therefore estimated that each SUB and INF sample would need to be sequenced across the equivalent of 1 HiSEQ lane (∼300 million reads) to facilitate robust comparative analyses of *Epichloë* gene expression. STR samples were assigned one-third of an HiSEQ lane each, as the fraction of *Epichloë*-derived reads was expected to be much higher in STR. Samples were submitted to the Iowa State University DNA Facility for 150 nt single-ended read mRNA sequencing on a HiSEQ 3000 system (Illumina, San Diego, CA, USA), with libraries prepared using the TruSeq RNA Library Prep Kit V2 (Illumina) as per the manufacturer’s instructions. Where required, additional sequencing was performed to bring the number of *Epichloë* reads for each sample to comparable levels; however, this was not reasonably achievable for Eel-INF samples, as the number of *Epichloë*-derived reads in these samples was very low (Supplementary Table 1). In total, these libraries were sequenced across 19 HiSEQ lanes, with the distribution of samples across these lanes described in Supplementary Table 1.

### Identifying core gene sets for STR vs SUB and INF vs SUB tissues

Gene annotations were created for each *Epichloë* species using the RNAseq data combined with previously published genomes for *E.* *typhina* strain E8 and *E.* *festucae* strain E2368 (Schardl *et al.* 2013). A draft genome for *E.* *elymi* strain NFe728 (Bioproject PRJNA623950) was assembled de novo using SPAdes version 3.11.1 (Bankevich *et al.* 2012). Transcripts for each *Epichloë* species were identified by aligning RNAseq reads from each sample to their respective reference genomes using STAR version 2.5.2 (Dobin *et al.* 2013). The aligned RNAseq data were then used to identify putative transcripts using the Funannotate v1.5.3 pipeline (https://github.com/nextgenusfs/funannotate; last accessed 4 March 2022), which makes use of AUGUSTUS v3.3.1 (Stanke *et al.* 2008) for gene calling. In all cases, the previously published *E. festucae* M3 gene models (Schardl *et al.* 2013) were used as evidence for training gene models. High-throughput functional annotation of gene models was performed using PANNZER2 (Törönen *et al.* 2018). The results were used as gene model sets for each species, which were assembled into orthologous groups (orthogroups) across species using proteinortho v5.0 (Lechner *et al.* 2011) and assigned unique identifiers with an “og_” prefix (Supplementary Table 2).

Prior to differential expression (DE) analysis, RNAseq reads for each sample underwent quality control using fastqc v0.11.8 (Andrews 2014), and adapter and poor quality reads removed with trimmomatic v0.38 (Bolger *et al*., 2014). To find expression levels of each gene, read counts of each sample aligned against their respective *Epichloë* gene model set were estimated with Salmon v0.13.1 (Patro *et al.* 2017). The count data were imported into R v3.6.0 (R Development Core Team 2013) using tximport v1.10.1 (Soneson *et al.* 2015). For each *Epichloë* species, genes that were differentially expressed between samples of different tissue types (e.g. STR and SUB) were identified with R package DESeq2 v1.22.2 (Love *et al.* 2014). In order to reduce spurious results, DE estimates with high variance between samples or low read counts were minimized using R package apeglm v1.4.2 (Zhu *et al.* 2019).

A core set of genes exhibiting a minimum 2-fold change in expression in STRvSUB, STRvINF, and INFvSUB tissues were identified using R v3.6.0, with an *S*-value (Stephens 2017) cutoff of ≤0.005 used to account for multiple testing when identifying genes with significant differences in expression. To assess the significance of the observed number of orthologous genes with shared DE trends, DE values were permuted within the transcriptomes of each species and the permuted data set was tested for core genes 10,000 times to predict the frequency at which orthologous genes with shared DE trends would be expected to occur by chance. The functional predictions for proteins encoded by the core-STRvSUB and core-INFvSUB DEGs were manually refined using data from InterProScan (Jones *et al.* 2014), FungiDB (Basenko *et al.* 2018), and NCBI BLAST (Johnson *et al.* 2008).

### Gene cluster identification

Gene clusters were identified as contiguous groups of at least 3 genes with DE of 2-fold or more in the same direction and an *S*-value (Stephens 2017) cutoff of ≤ 0.01. Clusters with 4 or more genes were allowed 1 “gap” gene with less than 2-fold DE. Putative biosynthetic gene clusters (BGCs) were identified through manual review of the encoded protein sequences, and all gene models from STRvSUB BGCs were manually reviewed against aligned RNAseq reads, with adjustments made as required.

### Comparative genome analysis

A subset of the multitude of sequenced *Epichloë* genomes was selected to represent sexual and asexual isolates across the evolutionary and ecological diversity of the genus *Epichloë* (Supplementary Table 10). Selected sexual genomes were limited to isolates for which stroma formation had been unambiguously observed, whereas putative asexual genomes were selected from nonhybrid isolates for which only seed transmission had been reported. These genomes were analyzed using BUSCO docker container busco: v4.1.1_cv1in the genome mode (Simão *et al.* 2015; Waterhouse *et al.* 2018), and those with anomalously poor scores or high rates of gene duplication (suggesting hybridization) were omitted from this analysis (Supplementary Fig. 5). The Eel, Efe, and Ety gene models from the core-STRvSUB gene set were manually curated using mapped RNAseq reads to remove modeling errors made by the automated pipeline, most commonly involving the erroneous addition of introns at the 5′ or 3′ ends of genes. The corrected Eel core-STR models were then used to represent the core-STR gene set as query sequences for blastn 2.6.0+ (Camacho *et al.* 2009) analysis of the selected genomes, with the exception of og_2625, og_4571, and og_4927, which were represented by the Efe orthologs due to the Eel sequences being split across contigs. The top blastn hit with an E-value < 5 was extracted from each genome and aligned using MUSCLE 3.8.31 (Edgar 2004), with the aligned sequences being manually reviewed and extended where possible to include as many full-length gene sequences as the genome assemblies allowed. In some cases, gene model start codon locations were further refined based on these alignments. The finalized alignments are available in the file “Supplementary alignments.zip.” These alignments were analyzed to determine if each gene in each isolate was missing, present as a partial sequence, present but split across contigs due to incomplete assembly, or present as a full-length homolog. CDS alignments of all full-length homologs for each gene were further analyzed to identify any sequences containing premature stop codons (relative to the reference gene model) or frameshift-causing insertions/deletions. These sequences were annotated as pseudogenes, except for sequences containing premature stop codons that were not more than 100 bp or 10% of the total CDS length (whichever was shorter) upstream of the reference stop codon location.

### PCR screening of og_0042

Amplification of og_0042 from *Epichloë* genomic DNA templates was performed using primers og_0042_F (ACCCTGAAGGCGAATGTTAC) and og_0042_R (GCCGACCTCGACGCCAAATG) with GoTaq DNA polymerase (Promega), following the manufacturer’s instructions for a 30 cycles amplification with an annealing temperature of 56°C. Template integrity control amplifications targeting the *tefA* gene were performed under the same conditions using the primers tef1-exon1d-1 (GGGTAAGGACGAAAAGACTCA) and tef1-exon6u-1 (CGGCAGCGATAATCAGGATAG) (Moon et al. 2002).

### Confocal laser-scanning microscopy

Stromata (STR) and substromal stem (SUB) samples were cut in approximately 0.5 cm cross sections by hand with a scalpel blade. The samples with wheat germ agglutinin-Alexa Fluor 488 Conjugate (ThermoFisher Scientific, Massachusetts, USA) and Aniline Blue diammonium salt (Sigma-Aldrich Chemie GmbH, Traufkirchen, Germany) as previously described (Becker *et al.* 2018). Sections of 100 µm were taken with a vibration microtome (Thermo Scientific, Microm HM 650V) cutting an approximately 1 cm^3^ block of 5% agarose (Agarose SERVA Low Melting; SERVA, Heidelberg, Germany) containing the pre-stained samples. The 100 µm slices were transferred to a slide and embedded in staining solution for 10 min before confocal laser-scanning microscopy was performed as previously described (Becker *et al.* 2018).

### Extraction and preparation of samples for measurement of natural product concentrations

Freeze-dried STR, SUB, and INF samples were harvested from a range of different *Epichloë* associations (Supplementary Table 9) following the onset of host flowering following a period of natural or artificial vernalization. Approximately 50 mg of freeze-dried material from each sample was then ground to powder and extracted in 50% (v/v) methanol in water, with the volume of the extraction solvent adjusted to a final concentration of 50 mg plant material/ml. Samples were extracted at room temperature with 30 RPM end-over-end rotation in the dark for 1 h, centrifuged for 10 min at 16,000 × *g* to remove particulate matter, then transferred to amber glass HPLC vials through a 13 mm diameter, 0.45 µm pore polytetrafluoroethylene syringe filter (Jet BioFil, Guangzhou, China).

### Analysis of indole diterpene and ergot alkaloid concentrations

Samples (5 µl injection) were chromatographically separated on a Kinetic C18 150 × 2.1 mm (2.6 µm) column (Phenomenex, Torrance, CA, USA) using the following linear gradient profile (eluent A, aqueous 0.1% formic acid and eluent B, acetonitrile with 0.1% formic acid); time 0 min (T0) at 10% B, T6 at 60% B, T17 at 100% B, and T19 at 100% B, followed by equilibration to initial conditions over the following 8 min. Detection and quantitation was achieved using a QTRAP 6500+ (AB Sciex LLC, Framingham, MA, USA) using ESI in positive ion mode and a nebulizer temperature of 400°C. Instrument parameters common to all analytes were: curtain gas 25, ionspray voltage 3750 V, ion source gas 1 and 2 both 60, entrance potential (EP) 10 V, with a 0.5-s cycle time. Supplementary Table 11 shows the parameters specific to each compound.

The raw data was processed using MultiQuant v3.0.2 (AB Sciex LLC) to integrate and determine peak areas for target compounds. For the paspaline, paxilline, and terpendole derivatives, quantitation was achieved by comparison of the peak areas to a paxilline standard (220 ng/ml) and are reported as paxilline standard equivalent (µg/g DM). For the lolitrems, quantitation was achieved by comparison of the peak areas to a lolitrem B standard (1.2 ng/ml) and are reported as lolitrem B standard equivalent (µg/g DM).

### Analysis of pyrrolopyrazine and pyrrolizidine concentrations

Each sample (1 µl injection) was chromatographically separated on a Poroshell HILIC-Z 150 × 2.1 mm (2.7 µm) column (Agilent Technologies, USA) with a flow rate of 300 µl/min using a linear gradient profile (eluent A is aqueous 16 mM ammonium formate and eluent B is 97% acetonitrile with 0.1% formic acid) with time 0 min (T0) at 92.8% B, T10 at 64.8% B, T11.5 at 64.8% B, and T12 at 92.8% B, followed by equilibration to initial conditions over the following 3 min. Analytes were detected using a Q-Exactive orbitrap mass spectrometer (Thermo Fisher Scientific, Waltham, MA, USA) equipped with a HESI probe run in positive ion mode with the capillary temperature at 275°C and a spray voltage of 2.5 kV. Gas flow rates were as follows: sheath gas 13, auxiliary gas 1, sweep gas 1. Ions were collected between 80 and 300 m/z at 35,000 resolution. Supplementary Table 12 details the retention times and ions of the analytes and internal standard.

The raw data was processed using LCQuan 2.7 (Thermo Fisher Scientific). For peramine, cyclo(Pro, Arg) and the lolines (NAL, NANL, NFL), LCQuan was used to determine the final concentration in the samples. For the cyclo(OH-Pro, Arg), cyclo(Pro, Me-Arg) and cyclo(OH-Pro, Me-Arg), where standards were not available, concentration was estimated based on the response factor for cyclo(Pro, Arg).

## Results

### Experimental design, microscopy, and RNAseq statistics

**Fig. 1.**
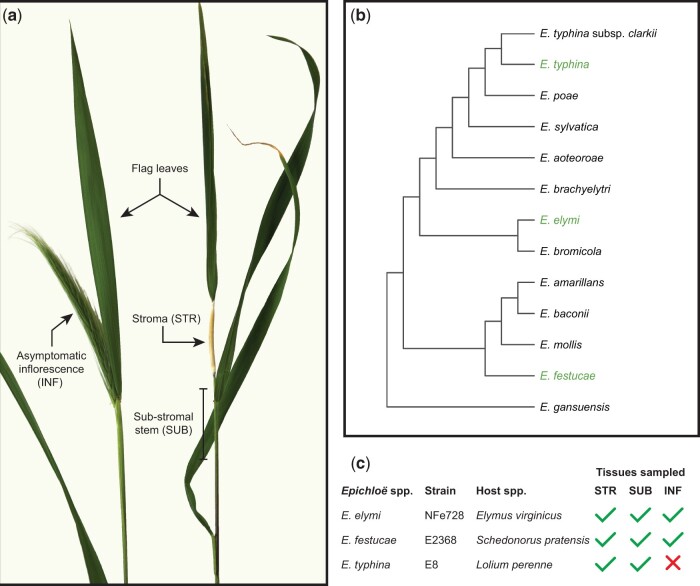
Experimental design. a) Photo showing *E. elymi*-infected *Elymus virginicus* asymptomatic (left) and a symptomatic (right) reproductive tillers annotated with the locations of sampled tissues. Tillers shown are of advanced age relative to the actual time of sampling. b) Phylogenetic tree of representative *Epichloë* species. c) Summary of sample types harvested for this study; Ety-INF samples were not available as Ety forms stromata on every infected tiller.

**Fig. 2.**
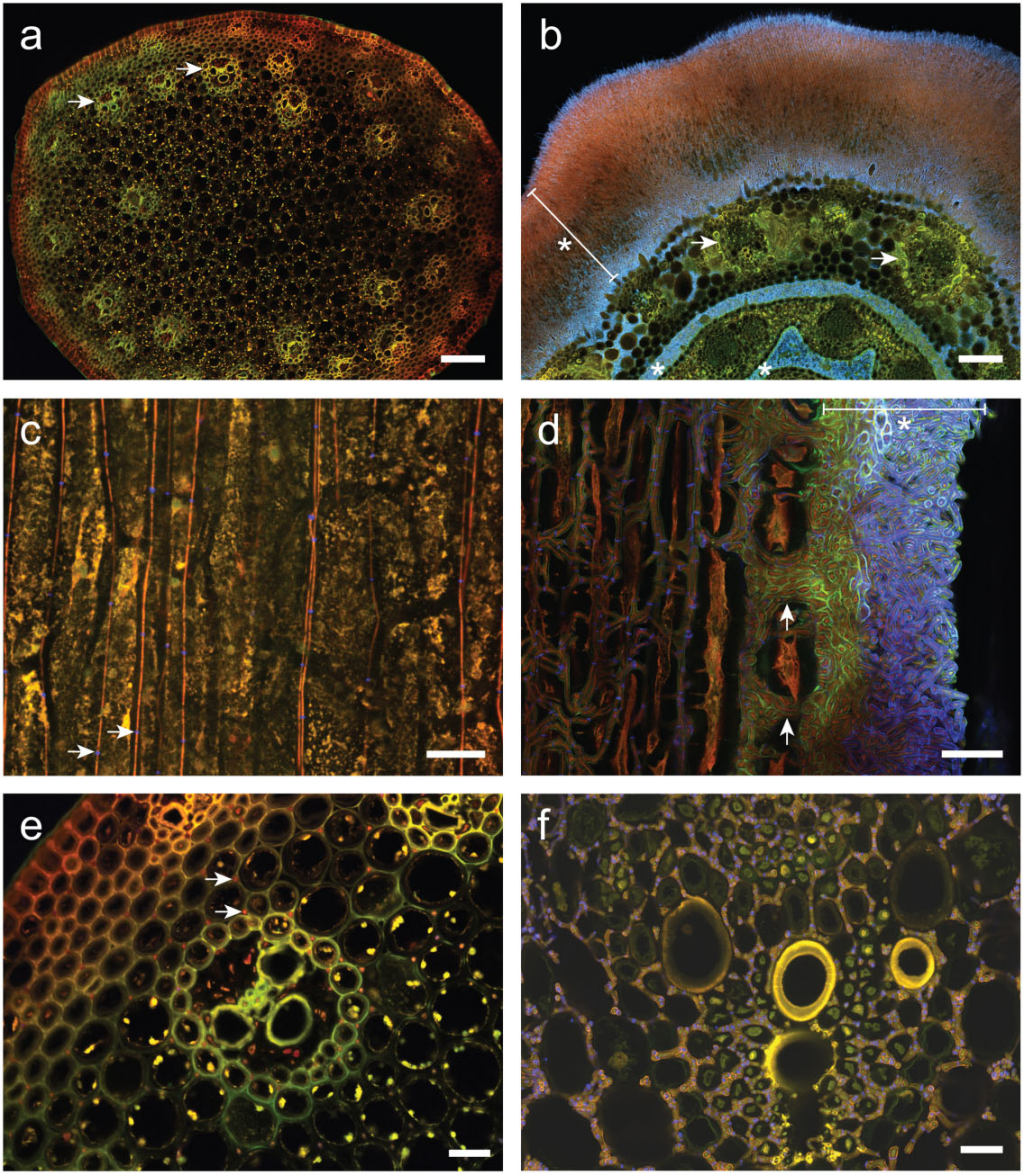
Microscopic imaging of symptomatic *Elymus virginicus* reproductive tillers demonstrates the virulent growth of *E. elymi* within stroma tissues. Samples were treated with wheat germ agglutinin-Alexa Fluor 488 (WGA-AF488) conjugate and aniline blue. WGA-AF488 stains chitin (captured as blue pseudocolor) present in the septa of endobiotic hyphae, and the septa and cell wall of epibiotic hyphae. Aniline blue stains β-d-1,3-glucans in both plant and fungal cell walls (captured as green pseudocolor). Autofluorescence of chlorophyll and the fungal cytoplasm is captured as red pseudocolor, merging to orange where this signal overlaps with green. Scale bar lengths are 100 µm (a, b) and 20 µm (c, d, e, f). Images (a) and (b) show transverse sections (perpendicular to the stem axis) for asymptomatic infected stem tissues harvested from 2 to 3 cm below the base of a stroma (a) and from unfertilized stroma tissues (b). Arrows in (a) and (b) highlight examples of host vascular bundles. The dense layers of epibiotic *E. elymi* hyphae that separate and envelop host tissues are indicated on (b) using asterisks (*) and a capped line that spans the external hyphal layer. Images (c) and (d) show longitudinal sections of the asymptomatic stem (c) and stroma (d). Arrows in (c) highlight examples of septa (blue) on endobiotic *E. elymi* hyphae (vertical orange lines). Arrows in (d) show where hyphae have burst through the host epidermis during stroma formation, with the external hyphal layer indicated by an asterisk and capped line. Images (e) and (f) show close-ups of host vascular bundles in transverse sections from asymptomatic stem (e) and stroma (f) samples. Arrows in (e) show examples of individual *E. elymi* hyphae occupying the intercellular spaces between host cells. Note the virulent hyphal growth and loss of cohesion for the vascular bundle contained within the stroma sample (f).

We performed RNAseq experiments on 3 different *Epichloë*-host associations, including *E.* *virginicus* infected with *E.* *elymi* strain NFe728 (Eel), *S.* *pratensis* (syn. *Festucae pratensis*, *Lolium pratense*) infected with *E.* *festucae* strain E2368 (Efe), and *L.* *perenne* infected with *E.* *typhina* strain E8 (Ety). All *Epichloë* strains analyzed in this study have haploid genomes. We compared expression levels between emerging stromata (STR) and an asymptomatic section of the plant’s reproductive stem taken from 1 to 5 cm below the base of each stroma (SUB) (Fig. 1, a and c). Despite their close proximity on host reproductive tillers, the physiologies of these STR and SUB samples were highly distinct when observed macroscopically (Fig. 1a; Supplementary Fig. 1). Confocal laser-scanning microscopy revealed the virulent growth of Eel hyphae in STR samples, including the hyperproliferation of both epibiotic and endobiotic hyphae, and extensive invasion and loss of coherency within host vascular bundles (Fig. 2). In contrast, Eel growth within SUB tissues was highly restricted, and host vascular bundles retained their cohesion (Fig. 2). Given the close proximity and common origins of the hyphae from paired STR and SUB samples, we concluded that *Epichloë* gene expression in SUB tissues would provide a suitable asymptomatic baseline for comparing with virulent gene expression in STR samples. Asymptomatic infected inflorescences (INF) were also sampled for the Eel and Efe associations, which formed stromata at intermediate frequencies. INF could not be sampled for the Ety association as this antagonistic *E. typhina* strain forms stromata on every infected reproductive tiller. All samples were harvested from a single plant of each association to eliminate variation driven by the host genotype, and the 3 selected *Epichloë* species are well-distributed across the evolutionary diversity of this genus (Fig. 1b). Thus, this experiment was designed to allow us to differentiate the core genes associated with stroma formation from changes that are specific to a particular *Epichloë* species, host species, or association.

RNA was extracted and sequenced from 3 samples per tissue type per association, and the resulting RNAseq reads were mapped against gene models for the corresponding *Epichloë* species. RNAseq reads from SUB tissues contained an *Epichloë* component of 1.4–1.7% in Eel samples, 3.3–5.4% in Efe, and 2.6–3.5% in Ety (Supplementary Table 1). This low percentage of *Epichloë* reads was expected because most RNA molecules extracted from asymptomatic *Epichloë*-infected grass tissues are derived from the host plant (Dupont *et al.* 2015; Schmid *et al.* 2017; Nagabhyru *et al.* 2019). The *Epichloë* component of STR reads was much higher, at 29–35% for Eel, 15–20% for Efe, and 20–28% for Ety, consistent with the massive increase in *Epichloë* hyphae observed in these samples (Fig. 2). Interestingly, although the *Epichloë* component of Efe-INF reads (2.7–6.4%) was similar to that of Efe-SUB, the *Epichloë* component of Eel-INF reads (0.05–0.08%) was much lower than in Eel-SUB tissues. This suggests that *E. elymi* may colonize INF tissues less extensively than *E. festucae*, or that *E. elymi* reads are being diluted by the relatively increased plant mass of *E. virginicus* inflorescences compared with *S. pratensis*.

### Comparative analysis defines the core differentially expressed gene sets

**Fig. 3.**
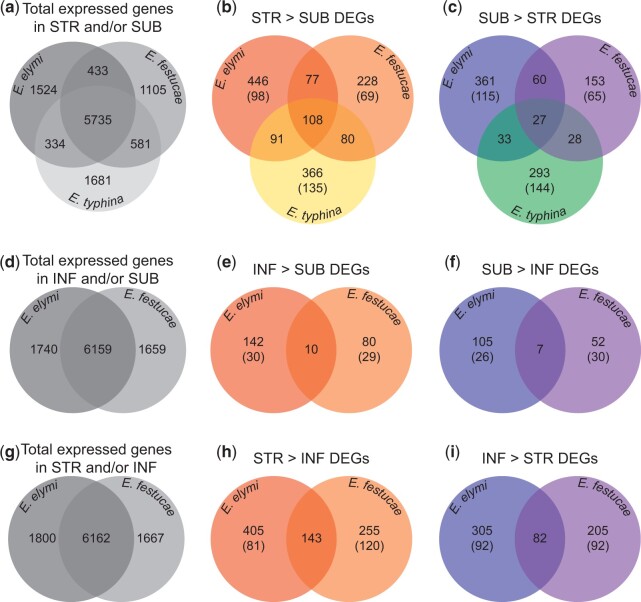
Comparative analysis across 3 associations defines the core-STRvSUB and core-INFvSUB DEG sets. The top row (a–c) shows comparisons of the STRvSUB analyses across all 3 associations, while the middle row (d–f) and bottom row (g–i) show comparisons of the INFvSUB and STRvINF analyses, respectively, from the Eel and Efe associations (INF are not produced in the Ety association). a), d), and g) Distribution of expressed gene models utilized during this study. The central panels show the distribution of DEGs identified in the STRvSUB analysis (b, c), the INFvSUB analysis (e, f), and the STRvINF analysis (h, i). Values in parentheses show the number of DEGs that were identified as species-specific, meaning that they lacked an ortholog in the genomes of the other *Epichloë* species being compared.

We first identified *Epichloë* genes that were differentially expressed between STR and SUB to gain insight into the transcriptome changes associated with the proliferative growth of *Epichloë* hyphae in STR relative to the restricted growth in SUB (Fig. 2). Each association was analyzed independently, with the minimum definition of a differentially expressed gene (DEG) set at a statistically significant (*S*-value < 0.005; Stephens 2017) 2-fold change in expression for all analyses. In total, we identified 1203 Eel-STRvSUB DEGs (722 STR > SUB and 481 SUB > STR), 761 Efe-STRvSUB DEGs (493 STR > SUB and 268 SUB > STR), and 1026 Ety-STRvSUB DEGs (645 STR > SUB and 381 SUB > STR) (Fig. 3; Supplementary Table 2). In total, 15% of all expressed genes were identified as DEGs in Eel, while 10% were DEGs in Efe and 12% were DEGs in Ety.

The per-association STRvSUB DEG sets were referenced against an index of orthologous gene models to identify the conserved set of transcriptional changes that differentiate these tissues across all 3 *Epichloë* associations. This “core” STRvSUB set totaled 135 genes, including 108 STR > SUB DEGs and 27 SUB > STR DEGs (Fig. 3; Supplementary Table 3). Permutation analysis revealed that the number of core-STRvSUB DEGs observed greatly exceeds predictions assuming random distribution of DE (Supplementary Fig. 2), suggesting the differential expression patterns of these genes have been maintained since the divergence of these 3 *Epichloë* species. The majority of STRvSUB DEGs that were not represented in this core set were either association-specific genes or genes that exhibited association-specific differential expression, with a smaller number of DEGs being shared between only 2 of the 3 species (Fig. 3). STR > SUB DEGs were more prevalent than SUB > STR DEGs in the per-association DEG sets, suggesting that activation of gene expression contributes more to STR differentiation than repression of gene expression, and this trend was even stronger in the core-STRvSUB set.

We also compared the INF and SUB datasets to identify INFvSUB DEGs in Eel and Efe. Ety-INF tissues were not available for an equivalent analysis because *E. typhina* stromatized all infected *L. perenne* flowering tillers. Overall, the total number of INFvSUB DEGs were much lower than STRvSUB DEGs, with only 264 Eel-INFvSUB DEGs identified (152 INF > SUB and 112 SUB > INF) and 149 Efe-INFvSUB (90 INF > SUB and 59 SUB > INF) (Fig. 3; Supplementary Table 4). These Eel-INFvSUB and Efe-INFvSUB DEG sets also were compared with identify the core set of INFvSUB DEGs shared by both species, which contained only 10 INF > SUB and 7 SUB > INF DEGs (Fig. 3; Supplementary Table 5). This observed number of core-INFvSUB DEGs again exceeded the number predicted assuming randomized distribution of differential expression, though not as dramatically as observed for core-STRvSUB genes (Supplementary Fig. 2).

Previous studies compared gene expression in symptomatic/stromatized inflorescence tissues (STR) with asymptomatic infected inflorescence tissues (INF), reasoning that, based on the associated host tissue types, INF tissues may represent the most direct counterpart to STR (Zhang *et al.* 2010; Wang *et al.* 2019). The absence of Ety-INF samples precluded the use of an equivalent analysis across all 3 associations investigated in this study. Nevertheless, we generated and compared STRvINF datasets for the Eel and Efe associations to investigate how comparable this STRvINF analysis is to our STRvSUB analyses. In total, we identified 935 Eel-STRvINF DEGs (548 STR > INF and 387 INF > STR) and 685 Efe-STRvINF DEGs (398 STR > INF and 287 INF > STR) (Fig. 3; Supplementary Table 6). Comparison of these Eel- and Efe-STRvINF DEG sets identified 225 core-STRvINF DEGs that exhibited conserved differential regulation between both associations (143 STR > INF and 82 INF > STR), greatly exceeding predictions assuming random DEG distribution (Supplementary Fig. 2).

Analysis of the expression patterns observed in the STRvINF analyses showed they were reasonably similar to the STRvSUB analyses. For the Eel dataset, 349 genes were shared between the 722 STR > SUB DEGs and 548 STR > INF DEGs, while 229 genes were shared between the 481 SUB > STR DEGs and 387 INF > STR DEGs. Similarly, the Efe dataset contained 305 genes that were shared between the 493 STR > SUB and 398 STR > INF DEGs, while 153 genes were shared between the 268 SUB > STR and 287 INF > STR DEGs. Importantly, only 3 Eel genes (og_2207, og_11792, and og_4415) and 1 Efe gene (og_1508) exhibited contradictory STR-relative patterns of regulation in these STRvSUB and STRvINF analyses (i.e. the direction of regulation differed between the STRvSUB and STRvINF analyses when gene expression was compared using STR data as the numerator and SUB or INF data as the denominator). The correlation between the STRvSUB and STRvINF results was even stronger when considering core DEGs, with 95 of the 135 core-STRvSUB DEGs having overlapping entries in the core-STRvINF set that exhibited the same STR-relative pattern of regulation. Of the remaining 40 core-STRvSUB DEGs, 32 were identified as DEGs with the same STR-relative expression pattern in either the Eel-STRvINF or Efe-STRvINF DEG sets, but did not meet the DEG threshold in the second association. Finally, the remaining 8 genes were not identified as DEGs in either the Eel or Efe STRvINF analysis. Thus, the majority of core-STRvSUB DEGs exhibited the same STR-relative expression pattern in the STRvINF analyses, while the remainder were not identified as DEGs in the STRvINF analyses.

Of the 225 core-STRvINF DEGs, 130 lacked a counterpart in the core-STRvSUB DEG set. Orthologs to 25 of these 130 core-STRvINF DEGs were not found in Ety, precluding their appearance in the core-STRvSUB set. A further 90 of these 130 core-STRvINF DEGs exhibited an equivalent STR-relative expression pattern in the STRvSUB analyses from 2 associations (60 genes) or 1 association (30 genes) but did not reach the DEG threshold in the remaining association(s). Of the remaining 15 core-STRvINF DEGs, 11 were not identified as DEGs in any of the STRvSUB analyses. This left only 4 core-STRvINF DEGs that exhibited an inverted STR-relative expression pattern when compared with any of the 3 STRvSUB analyses. Furthermore, in each of these 4 cases the contrary results were limited to the Ety-STRvSUB dataset, with the Eel and Efe-STRvSUB datasets exhibiting the same STR-relative expression pattern as the core-STRvINF set. Collectively, these observations show that the results from STRvSUB analyses correlate strongly with STRvINF analyses. This suggests that despite STR and INF samples being found on the same host tissue (inflorescences), gene expression in INF samples is much more similar to SUB samples than to STR samples.

### Identification of coregulated gene clusters

The genes encoding biosynthesis of a fungal natural product are usually coregulated and organized into a contiguous BGC. To facilitate the discovery of BGCs that exhibit concerted expression changes in STR or INF tissues, which is described in detail within a later section, we first searched the STRvSUB and INFvSUB sets from each association to identify any contiguous cluster of coregulated genes that exhibited a minimum 2-fold change in expression (*S* < 0.01). The minimum definition for a cluster was set at 3 genes, with clusters of 4 or more genes allowed a single “gap” gene that did not meet the 2-fold differential expression requirement. This analysis identified 149 total STRvSUB DEG clusters across the 3 associations (Supplementary Table 7). This number was significantly higher than would be expected by chance (Supplementary Fig. 3); however, roughly one-third of these clusters are predicted to result from randomized DEG assortment rather than selective pressures. Each of the 149 clusters identified was given a unique number with clusters 1–51 being from Eel, 52–93 from Efe, and 94–149 from Ety (Supplementary Table 7). Of these 149 clusters, 84 were only identified in a single association, 28 were homologous clusters shared by 2 associations (12 Eel/Efe, 7 Eel/Ety, and 9 Efe/Ety clusters) and 3 were homologous clusters shared across all 3 associations (Supplementary Fig. 4). Therefore, 115 unique clusters were present within the 149 DEG clusters identified. Of the 28 homologous clusters identified, all but 2 exhibited the same STR-relative pattern of expression across their different host associations (Supplementary Fig. 4). It is important to note that this analysis only identified differentially regulated clusters; the failure to identify a cluster homolog does not necessarily reflect the absence of that cluster in the corresponding *Epichloë* genome. Homologous clusters almost always exhibited coregulation between associations, with the 2 exceptions being the homologous cluster pairs Eel-13/Efe-52 and Eel-20/Efe-75, for which the component clusters were regulated in opposite directions (Supplementary Fig. 4). A total of 11 INFvSUB DEG clusters were also identified, with 3 in Eel (clusters 150–152) and 8 in Efe (clusters 153–160) (Supplementary Table 8). However, none of these clusters were shared between Eel and Efe, and the number of clusters observed was within the upper limits of predictions assuming randomized DEG assortment (Supplementary Fig. 3).

### Functional analysis shows that expression of virulence-related genes is promoted and mutualism-related genes are suppressed in stromata

The 135 core-STRvSUB DEGs were assigned putative functional categories through extensive manual analysis and annotation based on existing literature for homologous sequences (Supplementary Table 3). Hyper-proliferation of endobiotic and epibiotic hyphae is a dramatic change associated with stroma formation (Fig. 2). Consistent with this observation, 11 core-STRvSUB DEGs (10 STR > SUB DEGs and 1 SUB > STR DEG) were identified as encoding proteins putatively associated with cell wall biosynthesis. These included genes associated with chitin biosynthesis, with STR > SUB DEGs including 2 genes encoding chitin synthase components (og_0362 and og_1935), and 1 gene encoding the enzyme glucosamine: fructose-6-phosphate aminotransferase (GFAT; og_5263), which catalyzes synthesis of the chitin precursor glucosamine-6-phosphate. Furthermore, the single SUB > STR DEG associated with cell wall biosynthesis (og_0021) encodes the enzyme glucosamine-6-phosphate deaminase, which catalyzes the reverse reaction to GFAT. This suggests that *Epichloë* species regulate chitin biosynthesis through transcriptional control of the enzymes responsible for fructose-6-phosphate ⇌ glucosamine-6-phosphate interconversion. The core-STRvSUB set also included 12 STR > SUB DEGs encoding proteins putatively associated with adaptation to the external environment, consistent with the huge increase in epibiotic hyphae during stroma formation. These included DNA repair enzymes associated with UV-induced damage and hydrophobins that form self-assembling layers at the hydrophobic–hydrophilic interface on the surface of hyphae and conidia. The proliferation of epibiotic hyphae is accompanied by the development of abundant conidiophores on the surface layer, which has single terminal conidia that act as spermatia for sexual crosses. The STR > SUB subset also contained 5 genes putatively associated with conidiation.

The synchronization of *Epichloë* stroma formation with the onset of host inflorescence development may enable the fungus to appropriate the associated influx of host nutrients to fuel its own sexual cycle. Seven STR > SUB and 4 SUB > STR genes encoding transporters were contained within the core-STRvSUB set. Besides the 3 transporter-encoding genes associated with natural product transport, these changes likely reflect the expansion of energy demand and changes to the energy sources utilized by stromata. *Epichloë* hyphae in stroma tissues are unique in that they extensively colonize host vascular bundles, which is accompanied by a very noticeable loss of cohesion between the host cells in these bundles (Fig. 2f). This invasive growth presumably facilitates access to the nutrients required to fuel proliferative growth. The upregulated core-STRvSUB set encoded 5 secreted proteins putatively associated with digestion of host structural biopolymers, which may be required to degrade host barriers impeding invasive colonization. These included a putative cuticle-degrading protease (og_1852), which could be specifically involved in forming the expressoria structures that are abundant in stromata (Fig. 2d). These proteins could also be involved in obtaining energy directly through digestion of host cell walls, though most grass cells contained within stromata appear to remain intact (Fig. 2). Enrichment of STR > SUB DEGs putatively associated with metabolism of aromatic and phenolic compounds was also observed, possibly in response to the increased availability of lignocellulose derivatives requiring digestion. Besides aromatic metabolism, other metabolism pathways represented within the STR > SUB DEGs included folate biosynthesis (og_3783, og_0437, and og_1865), which is required for growth by all organisms, and siderophore biosynthesis (og_5908). Iron homeostasis in *E.* *festucae* involves both the extracellular siderophore epichloënin A and the intracellular siderophore ferricrocin, which are synthesized by the NRPSs SidN and SidC, respectively (Johnson *et al.* 2013b; Forester *et al.* 2018). Both of these NRPSs incorporate the substrate N(5)-hydroxy-l-ornithine into their siderophore products, which is produced by the ornithine monooxygenase encoded by the gene *sidA* (Forester *et al.* 2018). Although the genes encoding SidN and SidC were not differentially regulated in STRvSUB, the *sidA* gene (og_5908) is an STR > SUB DEG, suggesting siderophore biosynthesis may be increased in stromata.

Previous RNAseq analyses have shown that the invasive and proliferative hyphal growth associated with stroma formation induces a defense response in plant cells (Wang *et al.* 2019), suggesting that the host reacts to *Epichloë* as a pathogen in stromata. Three of the putative effector-encoding *Epichloë* genes identified by Hassing *et al.* (2019) were core STR > SUB DEGs (og_0387, og_2151 and og_3044), with og_0387 previously identified as upregulated across 4 independent associations involving pathogen-like *E. festucae* mutants (Eaton *et al.* 2015; Chujo *et al.* 2019). This suggests that these effectors may suppress the host defense response to the virulent fungal growth observed during stroma formation. Also contained within this STR > SUB set was DEGs putatively encoding an extracellular DNase (og_3102) and an uncharacterized secreted histone lysine methyltransferase (og_1560). Histone-linked extracellular plant DNA has been shown to confer resistance against fungal plant pathogens (Wen *et al.* 2009), suggesting these proteins may be virulence factors responsible for interfering with or degrading such host defensive molecules. This was recently demonstrated for an extracellular DNase from the fungal maize pathogen *Cochliobolus heterostrophus* (Park *et al.* 2019).

The initiation of stroma formation is synchronized with host inflorescence development, resulting in the formation of a highly localized structure that is confined to the immediate proximity of the developing host inflorescence. In contrast, hyphae located in the reproductive stem immediately below each stroma exhibit a restricted growth pattern (Fig. 2) similar to the mutualistic *Epichloë* growth pattern observed during symbiosis with vegetative host tissues (Tanaka *et al.* 2006; Christensen *et al.* 2008). These features suggest the presence of fungal signaling pathways that respond to specific host flowering signals, resulting in the induction or modification of regulatory pathways required to establish stroma formation. Eight core STR > SUB genes that encode proteins putatively associated with signal transduction were identified, suggesting they may be associated with stroma-specific signaling. These pathways included G protein signaling (og_1769), lipid signaling (og_1739, og_3864), cation transport (og_1074, og_1562), and protein phosphorylation (og_4571, og_5085). One core SUB > STR gene associated with signal transduction through regulation of protein ubiquitylation was also identified (og_2711). Transcription factor (TF) genes were also well-represented within the core-STRvSUB set, with 5 STR > SUB and 5 SUB > STR TF genes identified. Six of these TF genes (og_0152, og_1748, og_2796, og_2977, og_3924, and og_5036) have homologs that have been functionally analyzed and shown to be involved in conidiation or perithecium formation in *Fusarium graminearum* (Son *et al.* 2011) and/or *Neurospora crassa* (Carrillo *et al.* 2017) (Supplementary Table 3). Given these processes are intimately associated with stroma development in *Epichloë* species, the differential expression of these characterized TF genes is likely responsible for regulatory changes required for stroma formation. Homologs of the TF-encoding STR > SUB DEG og_2625 and SUB > STR DEGs og_1602 and og_3071 were either not characterized or not present in the genomes of *F. graminearum* and *N. crassa*, whereas the homolog to the SUB > STR TF gene og_3360 has been functionally analyzed in both *F. graminearum* and *N. crassa* with no phenotype reported (Son *et al.* 2011; Carrillo *et al.* 2017). These uncharacterized TFs might also regulate genes for stroma development in *Epichloë* spp., or could be involved in regulating ancillary processes such as secondary metabolism.

*Epichloë* spp. are known to produce a range of natural products that provide their host plants with resistance against a wide variety of herbivores. As is typical for fungi, synthesis of each of these bio-protective molecules is encoded by a set of clustered secondary metabolism genes, known as a BGC. Several *Epichloë* BGCs for host-protective natural products are known to be strongly expressed in host vegetative tissues, but are not expressed in culture (Eaton *et al.* 2015; Chujo *et al.* 2019; Hassing *et al.* 2019). Of the 27 SUB > STR DEGs in the core-STRvSUB set, 4 belonged to BGCs that encode synthesis of known or suspected bio-protective metabolites. These included the NRPS-encoding *ppzA* gene (syn. *perA*; og_4348) and its adjacent transporter-encoding gene *mfsA* (og_0138), the ABC transporter-encoding gene *lgsD* (og_0975) from the *LGS* cluster, and a peptide cyclase-encoding gene (og_0146) from the *GIG* cluster. The NRPS PpzA has several different variants for synthesis of different pyrrolopyrazine products (Berry *et al.* 2019a). The PpzA-1 variant encoded by Eel and Ety produces peramine (Tanaka *et al.* 2005), which is a known insect feeding deterrent (Rowan *et al.* 1986), whereas the PpzA-2 variant encoded by Efe produces the diketopiperazine cyclo(Pro, Arg) (Berry *et al.* 2019a). The *mfsA* gene is divergently transcribed from and coregulated with *ppzA*; however, previous gene deletion and synteny analyses suggest the function of the encoded transporter may not be associated with PPZ biosynthesis (Berry 2011; Berry *et al.* 2019b). Interestingly, the core SUB > STR gene og_4412 encoded a putative proline-specific permease, making this a candidate importer for the pyrrolidine-containing amino acids substrates utilized by all PpzA isoforms (Berry *et al.* 2019a). The *LGS* cluster encodes synthesis of a leucine/isoleucine glycoside metabolite that is localized to the host apoplast (Green *et al.* 2020). This BGC is present across the Eel, Efe, and Ety genomes analyzed here, and contains at least 3 other genes that are coclustered with *lgsD*. These other *LGS* genes were generally also coregulated with *lgsD*, but were excluded from the core-STR set as they did not meet the *S* < 0.005 threshold in every association. The *GIG* cluster encodes proteins for synthesis of the epichloëcyclins, which are small cyclic ribosomally synthesized and post-translationally modified peptide (RiPP) natural products with suspected bio-protective properties (Johnson *et al.* 2015). The strongly expressed *gigA* gene encodes the peptide substrate (GigA) for epichloëcyclin biosynthesis, which contains an N-terminal signal peptide followed by a variable number of repeats separated by kexin protease recognition sequences (Johnson *et al.* 2015). Digestion of GigA liberates small peptides that are thought to be cyclized by the UstYA-like peptide cyclase encoded by the core SUB > STR gene og_0146, which is clustered with *gigA*. Closer analysis revealed that *gigA* also belongs to the SUB > STR DEG sets of all 3 associations, but was identified as belonging to different orthogroups in Ety (og_9613) vs Eel/Efe (og_6633) due to the high variability in *gigA* repeat content between these *Epichloë* species.

Besides the 4 genes from characterized BGCs described above, the core SUB > STR DEG set contained 11 genes (og_0059, og_0295, og_0984, og_1401, og_1417, og_1545, og_2248, og_3434, og_4412, og_4628, and og_5229) encoding proteins with predicted functions that could be involved in biosynthesis of bioprotective metabolites. However, analysis of their adjacent genes revealed that most of these genes do not form part of any coregulated putative BGCs, suggesting that their encoded proteins are more likely to be involved in other metabolic processes. The one exception was the NRPS-like protein encoded by og_3434, which contains a structure of sequential adenylation, thiolation, and reductase (A–T–R) domains that has previously been associated with biosynthesis of piperazine and isoquinoline alkaloid natural products (Forseth *et al.* 2013; Baccile *et al.* 2016). Four of the genes clustered with og_3434 also trend toward SUB > STR regulation, including a gene encoding a second A–T–R protein (og_3551), a cytochrome P450 monooxygenase (og_3644), an O-methyltransferase (og_3703), and a DUF302-containing protein (og_3607). This suggests that og_3434 is part of a BGC responsible for synthesis of a natural product with potential bio-protective properties.

Interestingly, 5 of the 6 core STR > SUB DEGs that were identified as being associated with secondary metabolism belonged to a single gene cluster. We previously described how this 5-gene cluster exhibited strong differential STR > SUB expression in *Dactylis glomerata* samples infected with *E. typhina* based on RT-qPCR analysis (Berry 2016). The genes in this cluster encode a predicted PLP-dependent transferase (og_1729), an NRPS-like A–T–R protein (og_1835), an isoflavone reductase-like protein (og_2004), an MFS transporter (og_2053), and an FAD-binding oxidoreductase/berberine bridge enzyme-like domain protein (og_2125). The functions of these proteins are consistent with biosynthesis of an amino acid-derived natural product, and the expression pattern of these genes suggests that any such metabolite would have a stroma-specific role. Interestingly, the central gene in this cluster (og_2004) was previously identified as 1 of only 32 genes that was universally downregulated across 4 associations involving pathogen-like *E. festucae* mutants (Eaton *et al.* 2015; Chujo *et al.* 2019). Furthermore, whereas all 5 cluster genes are not expressed in axenic culture of *E. festucae* (Hassing *et al.* 2019), og_2004 alone was expressed at reasonably high levels in the vegetative tissues of the *E. festucae/L.* *perenne* association. This gene was also expressed in all SUB and INF samples from this study, whereas expression of the remaining 4 genes from this putative BGC was negligible in those tissues. Such incomplete regulatory coordination is unusual for BGC genes, suggesting that og_2004 may also play a role during normal mutualistic growth.

### Expression of *Epichloë* genes associated with host invasion is promoted in asymptomatic inflorescences

Of the 10 core-INFvSUB genes that exhibited INF > SUB expression (Fig. 3f), 4 exhibited an equivalent STR > SUB pattern in either the Eel-STRvSUB or Efe-STRvSUB DEG sets, whereas all 7 core SUB > INF DEGs were also members of the Eel-SUB > STR and Efe-SUB > STR sets. However, there was no overlap between core-INFvSUB and core-STRvSUB DEGs. The core INF > SUB DEGs encode proteins with a range of predicted functions, including 3 TFs, 2 heat shock chaperones, 2 proteins of unknown function, a dehydrogenase, a predicted effector, and a secreted endo-polygalacturonase (PG) (Supplementary Table 5). Two of these genes, which encode a putative TF (og_6632) and an effector (og_6636), were not present in Ety, suggesting they may play INF-specific roles not required by *Epichloë* spp. that do not transmit vertically through the host seed. The PG encoded by og_6656 is a close homolog (58% protein sequence identity) to PG1 from the wheat pathogen *F. graminearum*, which is a virulence factor that works synergistically with xylanases to break down the host cell wall during invasion of cereal inflorescences (Paccanaro *et al.* 2017). Interestingly, the genes from each association that exhibited the largest INF > SUB expression bias (og_11792 in Eel and og_7115 in Efe) both encoded secreted beta-xylanases (BXs). Although these Eel and Efe xylanases were sufficiently divergent that they were not identified as orthologs by proteinortho, reciprocal blast analysis identified og_11792 and og_7115 as each other’s closest homologs, along with og_10267 from Ety, suggesting the xylanases encoded by these genes may be orthologous in function. The INF-specific expression of these PG and xylanase proteins suggests they may be required for infection of host inflorescences, similar to their counterparts in *F. graminearum* (Paccanaro *et al.* 2017). The 7 core SUB > INF DEGs were all localized 3 putative BGCs, which are described in greater detail in a later section. The absence of these 7 genes from Ety is likely the result of discontinuous distribution, which is a common occurrence amongst BGCs in *Epichloë* spp. (Schardl *et al.* 2013).

### Host-protective natural product concentrations exhibit significant but inconsistent differences between STR and SUB tissues

**Fig. 4.**
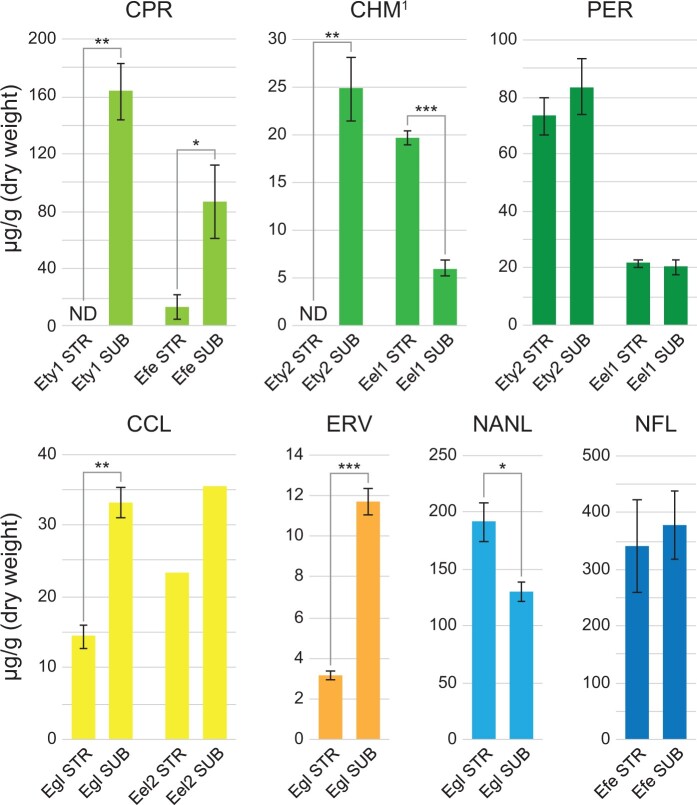
Host-protective natural product concentrations exhibit significant but inconsistent differences between STR and SUB samples. Illustrative examples of natural product concentrations from select associations are shown here; comprehensive results are available in Supplementary Table 9. Natural products shown include the pyrrolopyrazines cyclo(Pro, Arg) (CPR), cyclo(OH-Pro, Me-Arg) (CHM), and peramine (PER); the ergot alkaloids chanoclavine I (CCL) and ergovaline (ERV); and the loline alkaloids *N*-acetylnorloline (NANL) and *N*-formylloline (NFL). ^1^The concentrations reported for CHM are estimated based on the response factor of CPR, but the relative differences in CHM concentration are accurate. Values show the mean concentration of 4 samples for Efe, 2 samples for Eel2, and 3 samples for all other associations. Natural products that were not detected in any samples from a given set are labeled “ND.” Error bars show the standard error of the mean where *n* > 2. Means were compared between STR and SUB samples (where *n* > 2) using a 2-tailed *t*-test assuming equal variance (*P < 0.05; **P < 0.01; ***P < 0.001). Associations shown here include *E. elymi* E757/*Elymus virginicus* (Eel1); *E. elymi* NFe728/*E. virginicus* (Eel2); *E. festucae* E2368/*Schedonorus pratensis* (Efe); *E. glyceriae* (Lexington)/*Glyceria striata* (Egl); *E. typhina* (Switzerland)*/Dactylis glomerata* (Ety1); and *E. typhina* E8/*Lolium perenne* (Ety2).

In addition to the core-STRvSUB peramine and pyrrolopyrazine-specifying *ppzA* gene (og_4348) described above, genes from other well-characterized BGCs for production of known host-protective natural products exhibited strong SUB > STR expression bias. However, these BGCs were not represented within the core-STRvSUB set due to their discontinuous distribution across *Epichloë*. These included the 11-gene *LOL* cluster in Efe for production of the insecticidal loline alkaloids, and the minimalistic 4-gene *EAS* cluster in Eel for production of one of the simplest ergot alkaloid pathway products chanoclavine I. The genes in the Efe *LOL* cluster were strongly expressed in SUB and were almost silent in STR, consistent with a previous report that this pathway is downregulated in *E. festucae* stromata (Zhang *et al.* 2010). The same pattern was also observed for the Eel *EAS* cluster genes. Collectively, these results show that expression of known host-protective BGC genes is strongly repressed in STR tissues. STR, SUB, and some INF tissues were therefore harvested from a large range of *Epichloë*-infected grasses, and the concentrations of host-protective natural products was measured in these samples (Fig. 4, Supplementary Table 9). The results showed that while expression of the genes encoding synthesis of these host-protective molecules was extremely low in STR, this was not necessarily accompanied by a dramatic drop in STR metabolite levels. For example, ergot alkaloid levels were reduced in STR, whereas loline alkaloid levels were increased, and the peramine concentration was homogenous throughout the different sample types (Fig. 4, Supplementary Table 9). The mechanisms proposed to be driving these differences are addressed further in the Discussion.

### Identification of BGC candidates for synthesis of novel host-protective natural products

**Fig. 5.**
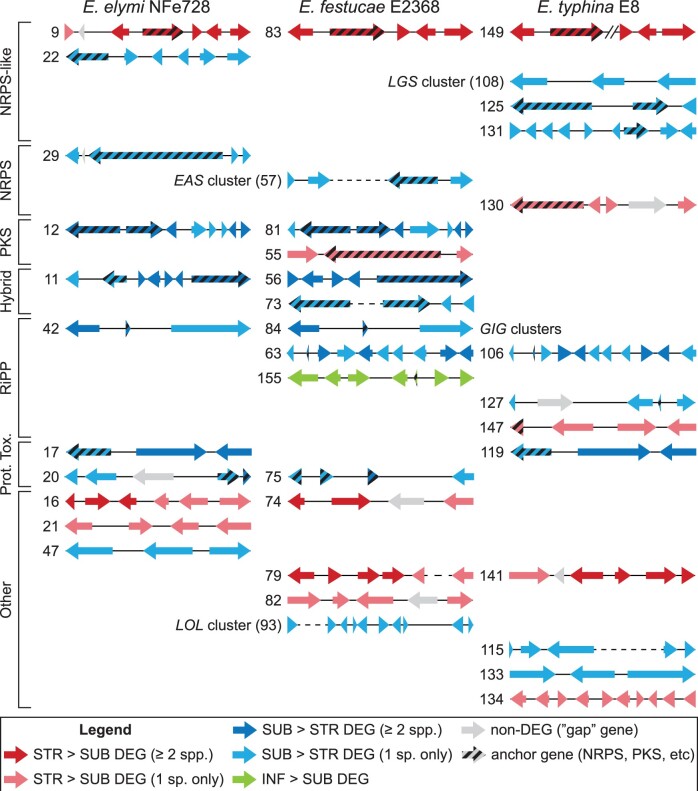
Putative biosynthetic gene clusters identified from the STRvSUB and INFvSUB analyses. Clusters not shown to scale. DEG clusters are grouped based on the class of “anchor” gene(s) they contain (NRPS, PKS, NRPS/PKS hybrid etc.). Clusters that encode several putative biosynthetic proteins but lack a canonical anchor gene are classed “other.” The previously characterized *LGS*, *EAS*, *GIG*, and *LOL* clusters are labeled in the figure. STR > SUB DEGs are shaded in red, SUB > STR DEGs are shaded in blue and INF > SUB DEGs are shaded in green. Homologous clusters that share at least 1 gene are put on the same row, with darker shading used to indicate the homologous DEGs within these clusters. Importantly, this figure only shows differentially expressed clusters; the absence of a cluster homolog for any species in this figure does not necessarily mean that cluster is missing from the corresponding genome. The diagonal lines in cluster 149 indicate the position of a contig break. Cluster numbering corresponds to the STRvSUB DEG clusters listed in Supplementary Table 6 and INFvSUB DEG clusters listed in Supplementary Table 7. STRvSUB clusters 17, 22, 56, 57, 63, and 83 were also identified in the INFvSUB analysis.

The observation that known host-protective natural product genes exhibited repressed expression in STR suggests that those uncharacterized BGCs also exhibiting SUB > STR expression profiles are candidates for encoding synthesis of novel host-protective natural products. Manual analysis of gene composition revealed that of the 115 unique DEG clusters identified during the STRvSUB analyses (Supplementary Table 7), 27 were likely to be BGCs that encode proteins involved in natural product or toxin biosynthesis (Fig. 5). These BGCs were defined where 1 or more genes encoded an “anchor” protein indicative of a particular natural product class, including polyketide synthases (PKSs), nonribosomal peptide synthetases (NRPSs), single module NRPS-like proteins, NRPS/PKS hybrids, protein toxins, and ribosomally synthesized and post-translationally modified peptide (RiPP) substrates. Clusters that lacked an anchor gene but predominantly encoded enzymes likely to be involved in natural product biosynthesis were also assigned as putative BGCs. To improve the accuracy of functional prediction, all gene models from these putative BGCs were manually reviewed against the aligned RNAseq data and corrected as necessary.

Of the 27 unique putative BGCs identified, 17 exhibited SUB > STR expression. These included the previously characterized *GIG* cluster in Eel and Efe, *LOL* cluster in Efe, and *LGS* cluster in Ety (Fig. 5). Components of the 11-gene Efe *EAS* cluster were also identified; however, expression of all Efe *EAS* genes was extremely low in both SUB and STR tissues, consistent with previous reports that this strain does not produce ergot alkaloids in planta (Schardl *et al.* 2013). Other known BGCs that were not identified in this analysis included the *EAS* cluster from Eel, the *GIG* cluster from Ety, and the *LGS* cluster from Eel and Efe. Closer analysis showed that the genes comprising these BGCs were universally downregulated in STR but were not identified as cluster components due to the fragmentary nature of the reference genomes in these regions. Similar problems are often encountered when investigating *Epichloë* BGCs due to their associations with subtelomeric regions and/or repetitive elements (Schardl *et al.* 2013). Given that all BGCs that are known to confer beneficial properties to the grass host exhibited SUB > STR regulation, the remaining 12 uncharacterized SUB > STR clusters may encode synthesis of novel natural products possessing activities that benefit the grass host. Also of interest was an unusual SUB > STR cluster of 5 (Eel cluster 33) or 6 (Efe cluster 66) genes encoding nonsecreted hypothetical proteins with no similarity to any previously characterized sequences (Supplementary Table 7).

Manual analysis of gene composition in the DEG clusters identified in the INFvSUB analyses (Supplementary Table 8) identified 1 SUB > INF Eel BGC, 1 SUB > INF Eel protein toxin cluster, 4 SUB > INF Efe BGCs, and 1 INF > SUB Efe BGC (Fig. 5). No overlap was observed between the BGCs identified within the Eel and Efe INFvSUB datasets; however, all SUB > INF BGCs were also identified as SUB > STR BGCs in the STRvSUB analyses of their respective associations (Fig. 5). In contrast, the 7 genes from the singular INF > SUB BGC (Efe cluster 155) were not identified as DEGs in the Efe-STRvSUB analysis. This cluster encodes proteins with functional predictions consistent with biosynthesis of an RiPP natural product, including 2 putative proline hydroxylases, 2 membrane proteins, a reductase, and a metallopeptidase (Supplementary Table 8). This cluster also contains 1 gene (og_7201) that is expressed at levels ≥ 100-fold higher than any other gene from the same cluster and encodes a small proline-rich 56 amino acid peptide with an N-terminal signal peptide, suggesting this gene encodes the RiPP substrate peptide. The unusual 6-gene cluster of hypotheticals described above (Eel cluster 33 and the homologous Efe cluster 66) (Supplementary Table 7) was also identified as the 5-gene cluster 157 in the Efe-INFvSUB analysis (Supplementary Table 8), though it was not as strongly downregulated as in Efe-STR. Most characterized *Epichloë* BGCs were not identified in this INFvSUB analysis, suggesting their regulation does not differ between INF and SUB tissues.

### DEGs upregulated in stromata are preferentially lost in haploid asexual *Epichloë* spp

Although the identification of differential gene expression associated with a specific condition suggests that these gene expression changes are important for the establishment or maintenance of that condition, this does not necessarily mean that the functions performed by these DEGs are specific to this condition. For example, while genes associated with chitin biosynthesis were expressed more strongly in STR vs SUB tissues, some expression of these genes is likely always required by *Epichloë* due to the essential nature of chitin biosynthesis during fungal growth. The prevalence of haploid asexual *Epichloë* strains that do not form stromata presented an opportunity to identify which core-STRvSUB DEGs are specifically involved in stroma formation, reasoning that these genes would be preferentially lost or functionally inactivated in asexual genomes. Therefore, sequences homologous to each core-STRvSUB DEG were extracted from the genomes of a diverse range of sexual and asexual nonhybrid *Epichloë* strains (Supplementary Table 10). Only genomes from haploid *Epichloë* strains that had been observed to produce stromata were selected to represent the sexual group, whereas the asexual group was represented by haploid seed-transmitted strains that have never been observed to form stromata. Asexual hybrid strains were excluded due to the complexities associated with the expected presence of multiple copies for most genes, as were genomes exhibiting low BUSCO assessment results suggesting poor quality assembly (Supplementary Fig. 5 and Supplementary Table 10). It is important to note that there is currently no conclusive way to prove that an *Epichloë* strain is asexual; the observation of a stroma proves that a strain can form stromata, but the absence of observed stroma formation does not prove that a strain cannot form stromata under any condition. Some of the strains designated asexual in this study may therefore be cryptic sexual strains, with this limitation being something we hoped to address using this comparative genome analysis.

**Fig. 6.**
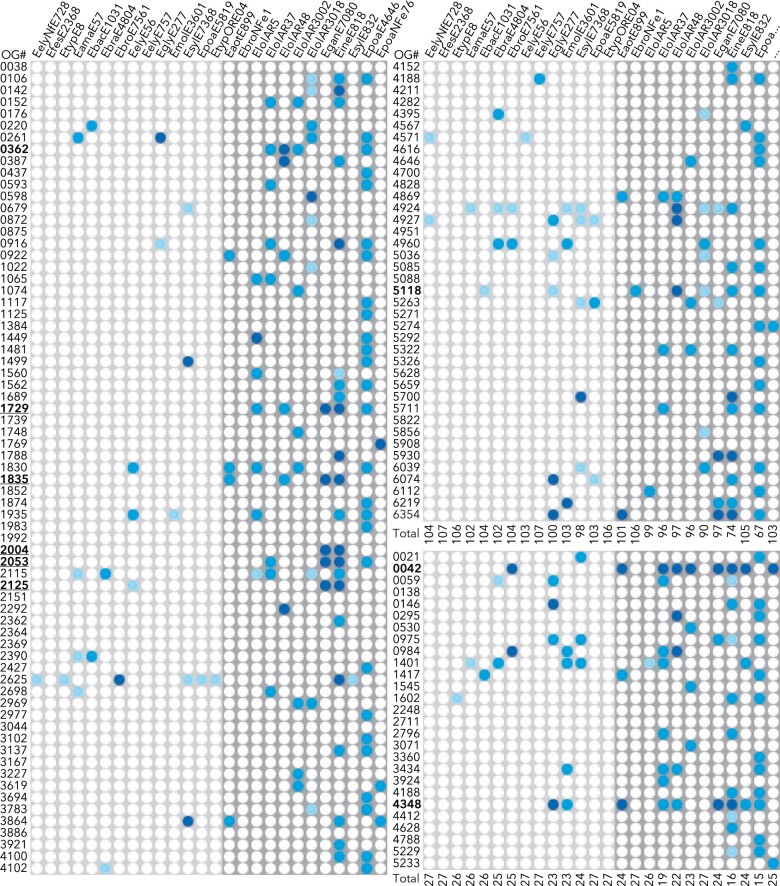
Comparative genome analysis identified core-STRvSUB genes that are preferentially lost or pseudogenized in asexual *Epichloë* spp. White circles indicate that the corresponding *Epichloë* genome contains a putatively functional copy of this gene, light blue circles indicate genes that are likely functional but are split across contigs, blue circles indicate pseudogenes, and dark blue circles indicate genes that are absent or mostly deleted in the target genome. Sexual *Epichloë* strains are indicated with a light gray background, and haploid asexual *Epichloë* strains with a dark gray background. STR > SUB DEGs are shown on the left and top right and SUB > STR DEGs are shown in the bottom right. The total number of putatively functional genes in each strain is summarized below each block. Orthogroup (OG) model numbers referenced in the text are shown in bold, with the 5 components of the only core-STRvSUB BGC being additionally underlined. The species and strains analyzed are shown along the top, with species abbreviated as follows: *E. amarillans*, Eama; *E. aotearoae*, Eaot; *E. baconii*, Ebac; *E. brachyelytri*, Ebra; *E. bromicola*, Ebro; *E. elymi*, Eely; *E. festucae*, Efes; *E. festucae* var. *lolii*, Elol; *E. gansuensis*, Egan; *E. glyceriae*, Egly; *E. inebrians*, Eine; *E. mollis*, Emol; *E. sylvatica*, Esyl; *E. typhina*, Etyp; *E. poae*, *Epoa*.

The extracted homologs for each core-STRvSUB DEG were aligned from start codon to stop codon. To minimize false negatives, all Eel, Efe, and Ety core-STRvSUB gene models were manually curated using mapped RNAseq reads to remove modeling errors made by the automated pipeline, most commonly involving erroneous extra introns, particularly at the 5′ or 3′ ends of genes. In some cases, the aligned sequences of core-STRvSUB gene models were used to further refine start codon locations. Each alignment was then analyzed to determine if a gene was putatively functional, present as a pseudogene, split across contigs, present as a partially deleted gene or absent from the genome of each strain (Fig. 6). The results showed that there was a general trend toward increased loss of core-STRvSUB DEGs in the putatively asexual strains, which retained an average of 88% core-STR genes compared with 98% for sexual strains (Fig. 6). There was some overlap between the ranges of retained core-STRvSUB genes in putative asexual (61–99%) and sexual strains (94–100%). In particular, the putatively asexual *E*. *bromicola* strain NFe1 retained all but 1 core-STR gene, suggesting it may retain the ability to form stromata. Strain genotypes were compared with identify any core-STRvSUB DEGs preferentially lost in putatively asexual species. On average, the number of sexual and asexual strains missing a functional copy of any given gene closely matched predictions assuming randomized distribution of loss among these core-STRvSUB DEGs (Supplementary Fig. 6). However, the loss rate in asexual strains was much higher than predicted for genes in orthogroup og_4348 (lost in 7/12 strains) and og_0042 (lost in 8/12 strains) (Fig. 6).

Orthogroup og_4348 contains alleles of the *ppzA* gene that encode NRPSs that synthesize pyrrolopyrazines, including the bio-protective natural product peramine (Tanaka *et al.* 2005; Berry *et al.* 2019a). However, this gene was also lost in 2 of the 14 sexual strains, and the discontinuous distribution of the *ppzA* gene across *Epichloë* is well-documented (Berry *et al.* 2015), suggesting that the high rate of og_4348 loss likely does not reflect an asexual-specific relaxation of selective pressure. In contrast, og_0042 is located within notoriously unstable AT-rich subtelomeric regions (Supplementary Fig. 7), which likely facilitates rapid loss of this gene after selection is relaxed (Schardl *et al.* 2013; Winter *et al.* 2018). Thus, the near-universal retention of this gene in sexual isolates and high prevalence of gene loss in asexual isolates suggests a strong sexual-specific selective pressure favoring retention of this gene, although this gene was missing from the genome of a single sexual isolate (*E. bromicola* E7561). Surprisingly for an apparently sexual-specific gene, og_0042 was the DEG that exhibited the highest SUB > STR expression bias in each of the 3 associations analyzed here, suggesting that strong repression of og_0042 expression is important for stroma development. The protein sequence encoded by og_0042 is approximately 450 aa in length and has no distinctive features or apparent homology to previously characterized proteins. We hypothesized that the high rate of og_0042 loss in asexual vs sexual isolates would make this gene an effective target for PCR screening of strain sexuality. The og_0042 multiple sequence alignment contained 2 highly conserved regions that were used to design primers for PCR amplification of an 810 bp gene fragment. A proof-of-concept screen was performed using available genomic DNA templates, which demonstrated amplification for 20 of the 21 stroma-forming isolates tested, and 1 of the 3 asexual isolates tested (Supplementary Fig. 8). Thus, the absence of an og_0042 amplification product using these primers can be used as an initial indicator for asexuality when screening haploid *Epichloë* strains.

Several other genes were absolutely conserved across the sexual *Epichloë* strains analyzed here but exhibited a relatively high rate of loss in asexual strains (Fig. 6). These included og_0362, which encodes a putative component of the chitin synthase complex 3 and was absent in 4/12 asexual strains, and og_5118, which encodes a putative amidoligase and was absent in 4/12 asexual strains. Finally, og_1729, og_1835, and og_2053, which all belong to the only upregulated BGC identified in the core-STRvSUB set (homologous clusters 9, 83, and 149; Supplementary Table 7), were absent or inactivated in 5/12, 4/12, and 4/12 asexual strains, respectively. Collectively, at least 1 of the 5 genes comprising this putative BGC was absent or inactivated in 7 of the 12 asexual strains analyzed here, whereas these genes were universally conserved in sexual strains. This strongly suggests an STR-specific role for any natural product associated with this putative BGC.

## Discussion

The initiation of stroma formation by *Epichloë* spp. represents a fundamental switch from the restricted asymptomatic growth of hyphae in host vegetative tissues to the virulent growth of hyphae that suppress host reproduction (Schardl and Leuchtmann 2005). The boundaries of the stroma structure are well-defined, being confined to the region of the host reproductive tiller containing the aborted host inflorescence. Our results demonstrate that stroma formation is accompanied by dramatic differences in *Epichloë* gene expression compared with the hyphae located in the asymptomatic reproductive stem immediately below each stroma. Previous RNAseq analyses of gene expression in stromata have focused on individual *E. festucae* associations (Zhang *et al.* 2010; Wang *et al.* 2019), meaning that the relevance of observed changes to other *Epichloë* species could not be determined. In contrast, we analyzed tissues across 3 different *Epichloë* species, which refined the much larger pools of species-specific gene expression changes to a core set of conserved changes that are proposed to be important for establishing and maintaining the asymptomatic and virulent growth phases exhibited by all sexual *Epichloë* species.

One important difference between this study and previous studies of stromata gene expression is that we used substroma stem (SUB) samples to reference gene expression changes in stromata (STR) samples, whereas Zhang *et al.* (2010) and Wang *et al.* (2019) used infected asymptomatic inflorescence (INF) samples for this purpose. Our choice was partly driven by practical requirements, as the Ety association does not produce any infected asymptomatic inflorescences, but we also wanted to understand how such a dramatic physiological differentiation between STR and SUB tissues can be achieved at such close proximity. Interestingly, only 49 of our 135 core-STRvSUB genes were identified in the STRvINF analysis of an *E. festucae*/*Festuca rubra* association by Wang *et al.* (2019); however, the relative direction of regulation (i.e. using STR data as the numerator) for these 49 DEGs was always identical to our own core-STRvSUB dataset. In contrast, 95 of 135 core-STRvSUB DEGs were also identified as DEGs in our own core-STRvINF dataset, all with the same relative direction of regulation. The absence of many core-STRvSUB genes from the dataset of Wang *et al.* (2019) appears to be due to a combination of factors. *Epichloë* sequence coverage was approximately 10-fold higher for our Efe SUB and INF datasets compared with the INF dataset of Wang *et al.* (2019), greatly increasing the relative statistical power of our own analysis. Compounding this lower sequence coverage, the methodology employed by Wang *et al.* (2019) required genes to meet a minimum RPKM threshold of 10 under both conditions to be included in the differential expression analysis. This resulted in genes that exhibited strong expression under 1 condition but had a mean RPKM value of <10 under the other condition, such as og_1729 and og_1835, were not identified as DEGs. In contrast, the methods we employed identified such situations as highly significant. We therefore conclude that the results from our STRvSUB analysis are highly comparable to STRvINF analyses, but we have also provided our own STRvINF analysis of the Eel and Efe associations to allow direct comparisons to be made.

Our microscopic imaging showed that STR samples are characterized by rampant proliferation of epibiotic hyphae to form a dense sheath. We also observed the hyper-proliferation of endobiotic hyphae and the invasion of host vascular bundles within STR tissues, presumably facilitating access to the host nutrients required to fuel stroma formation. Given their high biomass, we predict that the majority of *Epichloë* STR reads were derived from epibiotic hyphae. Consistent with this hypothesis we observed dramatically increased expression of genes associated with hyphal proliferation and adaptation to the external environment. However, we also saw signals that appear to come from tissues that constitute a smaller fraction of STR samples. These included genes that encode sporulation proteins that are presumably associated with the proliferation of conidia on the surface of STR, putative effectors that are likely secreted into the host apoplast by endobiotic hyphae to evade or suppress any host defense response, and putative digestive proteins that likely facilitate direct nutrient acquisition from host tissues by endobiotic hyphae. Protoperithecia had not formed at the early stage that STR were sampled, and in *Epichloë* spp. form only after fertilization, so the gene expression changes observed would not necessarily be expected to reflect similar studies on fruiting body gene expression in other fungi (Lehr *et al.* 2014; Trail *et al.* 2017). However, of the 5 core STR > SUB TF genes identified, 4 have homologs in *F.* *graminearum* and/or *N.* *crassa* reported to have deletion phenotypes related to sexual development (Son *et al.* 2011; Carrillo *et al.* 2017). Our analysis also identified 9 uncharacterized STR > SUB BGCs that may encode biosynthesis of natural products with roles in stroma formation and/or *Epichloë* sexual development, such as attracting the fly fertilization vector or inhibiting mycoparasites.

In contrast to the prolific hyphal growth observed in STR samples, our imaging showed that *E. elymi* colonization of SUB tissues occurs through restricted growth of predominantly endobiotic hyphae, consistent with previous observations of mutualistic *E. festucae* hyphae growing within host vegetative tissues (Tanaka *et al.* 2006; Christensen *et al.* 2008; Becker *et al.* 2016). This is reflected in the SUB transcriptomes of all 3 associations, which largely reflect the observations of previous studies that investigated *Epichloë* gene expression in vegetative host pseudostem tissues (Dupont *et al.* 2015; Eaton *et al.* 2015; Chujo *et al.* 2019; Hassing *et al.* 2019). These similarities between SUB and pseudostem transcriptomes are exemplified by the strong expression of all genes known to encode synthesis of host-protective natural products, including the loline alkaloid BGC in Efe, the ergot alkaloid BGC in Eel, and the pyrrolopyrazine gene *ppzA* found in all 3 species. All these host-protective genes also exhibited a very strong SUB > STR expression bias, suggesting that the 12 uncharacterized SUB > STR BGCs identified in this study represent strong candidates for specifying biosynthesis of novel host-protective natural products. Furthermore, the SUB > STR expression bias of genes specifying synthesis of the diketopiperazine-pyrrolopyrazines (Berry *et al.* 2019a), epichloëcyclin RiPPs (Johnson *et al.* 2015), and the Ile/Leu glycoside identified by Green *et al.* (2020) suggests that these natural products may also possess as-yet uncharacterized host-protective bioactivities. Putative hemolysin-like and Kp4/SMK-like protein toxins were also encoded within 2 SUB > STR gene clusters, and could also confer important bioprotective activities, similar to how the *Epichloë* Mcf protein toxin is thought to contribute anti-insect properties to the symbiosis (Ambrose *et al.* 2014). Given that the agricultural benefits and drawbacks of *Epichloë* spp. largely derive from the bioprotective properties conferred by their natural products (Schardl 2010), further investigation into the products specified by the putative BGCs described here would likely result in the discovery of novel *Epichloë* host-protective natural products and may provide important insights into the selection of *Epichloë* strains with desirable bioactivities for use in agriculture.

The mechanisms regulating transcription of BGCs specifying host-protective natural products remains an open question in *Epichloë*, as these are not expressed in culture (Tanaka *et al.* 2005; Young *et al.* 2005; Hassing *et al.* 2019), and it has been shown in both this and previous studies (Zhang *et al.* 2010; Wang *et al.* 2019) that these genes are not expressed in stromata. Of the 26 unique BGCs identified in this study, only 1 contained a gene encoding a putative TF (og_9937 in cluster 131), suggesting that unlike BGCs in many other fungi (Yu and Keller 2005; Keller 2019), *Epichloë* BGCs are generally not regulated by clustered TFs. The 5 SUB > STR TFs contained within the core-STRvSUB DEG set are therefore of particular interest, as they are candidates for regulating mutualistic processes such as the biosynthesis of bioprotective natural products. We speculate that further investigation may reveal 1 or more of these TFs to be a regulator of secondary metabolism in *Epichloë* species. We initially hypothesized that the global downregulation of host-protective genes in STR likely occurs to reduce or eliminate host-protective *Epichloë* natural products that may harm *Botanophila* flies and their larvae, which *Epichloë* spp. rely on as a fertilization vector to disseminate spermatia between stromata (Bultman *et al.* 1995, 1998). However, our results show that the relative metabolite levels in SUB and STR tissues are inconsistent, and likely largely depend on the mobility of the specific natural product. For example, the PPZ molecule peramine exhibited a homogenous distribution throughout the different tissue types, consistent with the proposed mobile nature of this metabolite (Koulman *et al.* 2007). In contrast, the reduced expression of *EAS* genes in STR resulted in a modest reduction in ergot alkaloid concentrations, suggesting that ergopeptines are relatively immobile within the plant. However, the reduction of ergot alkaloid concentrations in STR was much less dramatic than the reduction in *EAS* gene expression. This suggests that ergot alkaloids may have some mobility within the plant, or that the reduced *EAS* gene expression is compensated for by the huge increase in hyphal biomass. Indeed, loline alkaloid levels were even found to increase in STR despite the massive reduction in *LOL* gene expression. Thus, we conclude that the transcriptional repression of host-protective genes in STR does not serve to reduce or eliminate these molecules in stromata, but rather ensures the dramatic increase in hyphal biomass does not cause these metabolites to accumulate to high levels. The relative metabolite concentrations in STR and SUB tissues can also be used to gain insight into the mobility of uncharacterized *Epichloë* natural products. For example, the diketopiperazine-type PPZs cyclo(Pro, Arg) and cyclo(OH-Pro, Me-Arg) exhibited large yet inconsistent concentration differences in SUB vs STR samples from several associations. This suggests that, unlike peramine, the DKP-PPZs are immobile and are not transported out of the fungal cell, with their SUB vs STR levels instead being highly dependent on the degree to which a specific association represses expression of *ppzA* and any other relevant genes in STR.

Comparative analysis across a selection of haploid genomes from known sexual strains and suspected asexual *Epichloë* strains revealed that loss or pseudogenization of core-STRvSUB genes was generally more prevalent in asexual strains compared with sexual strains, though some overlap between these groups was observed. Most core-STRvSUB genes were conserved at similar rates between sexual and asexual genomes, presumably because many of the cellular processes upregulated in STR are not dispensable for growth in other tissues. However, there were some notable exceptions, including the 5 genes from the only STR > SUB BGC represented in the core-STRvSUB set. At least 1 of the 5 genes in this BGC was missing or pseudogenized in 7 of the 12 putative asexual genomes analyzed here, suggesting that the encoded biosynthetic pathway would be nonfunctional in most asexual isolates. In contrast, the 5 genes from this cluster were absolutely conserved across the 14 sexual genomes analyzed here. This strongly supports the hypothesis that this BGC encodes synthesis of a natural product with an important role related to stroma formation (Berry 2016). Another gene exhibiting a very strong trend toward loss in asexual strains was og_0042, which encodes an approximately 450 aa protein that has no significant similarity to any previously characterized protein sequences. This gene was missing from 8/12 asexual genomes, but only 1/14 sexual genomes, and was also the DEG that showed the strongest SUB > STR bias across all 3 associations analyzed here. This gene was also located in subtelomeric regions; for example, in the complete *E. festucae* Fl1 genome (Winter *et al.* 2018), og_0042 is the only gene annotated on a small GC-rich island contained within a notoriously unstable AT-rich subtelomeric region. This likely explains the abnormally high rate of gene loss over pseudogenization for og_0042 in asexual genomes. Given its extreme downregulation in STR and high rate of loss in asexual species that cannot or do not form stromata, we suggest that og_0042 may encode a repressor of stroma development that is quickly lost after unrelated mutations have accumulated to the point that a strain can no longer initiate its sexual cycle. The absence of this gene from the genomes of many asexual species also makes it a useful PCR target for indicating haploid strain sexuality.

We also investigated differential gene expression in INFvSUB samples in the Eel and Efe associations to better understand how these 2 sample types differ. Despite the proximity of the STR and SUB tissues, which were separated by only 1 cm on each reproductive tiller, gene expression in SUB samples was found to be much more similar to the INF samples taken from asymptomatic infected reproductive tillers. This finding is consistent with the restricted hyphal growth phenotypes of SUB and INF tissues relative to the proliferative hyphal growth observed in STR (Tanaka *et al.* 2006; Becker *et al.* 2016; Liu *et al.* 2017). Nevertheless, some differences between SUB and INF gene expression were observed. These included genes encoding a secreted endo-polygalacturonase (PG) and a secreted BX, which exhibited strong INF > SUB expression bias, suggesting they may be of specific importance for the colonization of host inflorescence tissues. This BX gene was also identified by Schirrmann *et al.* (2018) as 1 of 5 candidate *Epichloë* genes involved in divergent host specialization, providing an explanation for the high sequence divergence observed between the putative Eel, Efe, and Ety BX orthologs. The *Epichloë* PG protein is a close homolog of the polygalacturonase protein PG1 from the wheat pathogen *F.* *graminearum* that is known to work synergistically with xylanases to facilitate host colonization through plant cell wall digestion (Paccanaro *et al.* 2017), and in particular is implicated in facilitating invasion of host ovary tissues (Tomassini *et al.* 2009). We speculate that the *Epichloë* PG and BX proteins may similarly act synergistically to facilitate colonization of host inflorescences. The *Epichloë* genes encoding these proteins are therefore attractive targets for further study into *Epichloë* seed transmission, the high efficiency of which is important for the deployment of *Epichloë* strains in agriculture (Gagic *et al.* 2018). Referencing SUB samples also revealed other interesting nuances in INF gene expression. For example, our Efe-STRvINF analysis and the STRvINF analysis used by Wang *et al.* (2019) identified the genes from INFvSUB cluster 6 as being coregulated alongside the many other BGCs that are downregulated in STR, whereas our Efe-INFvSUB results show that this cluster is in fact unique in being strongly upregulated in INF tissues. This implicates a role for the putative RiPP product specified by this cluster in INF-specific processes, such as host seed colonization.

The fragmentary nature of the reference genomes used in this study directly resulted in the failure to rediscover several known *Epichloë* BGCs that exhibited strong SUB > STR expression bias, such as the Eel *EAS* BGC. It is therefore likely that aligning our RNAseq data against complete or near-complete *Epichloë* reference genomes, which can be assembled using modern long-read sequencing technologies (Winter *et al.* 2018), would have further increased the number of clusters identified. It is also notable that despite using current best-practice methods to prepare the Eel, Efe, and Ety gene models from RNAseq data, our manual revisions of the core-STRvSUB and STRvSUB BGC gene models revealed that approximately 30% of the gene models from this pipeline were incorrectly annotated. While a subset of these incorrect gene models involved alternate start codon locations that would be difficult to computationally predict without the aid of multiple sequence alignments, most cases involved the annotation of extra exons and introns that lacked supporting evidence in the RNAseq data. These erroneous annotations were predominantly located at the 5′ or 3′ ends of gene models, meaning that the conceptually translated sequences still contained most or all the actual protein sequence. The impact of these errors on our study are therefore predicted to be small. However, these results show that automated gene model annotation technology is still not fully mature. Manual revision of the models for any genes of interest is therefore recommended before commencing any follow-up studies.

In conclusion, the results presented here demonstrate that a set of evolutionarily conserved gene expression changes are associated with the characteristic asymptomatic-to-virulent switch that occurs during stroma formation by *Epichloë* species. By comparing 3 associations involving different *Epichloë* species we were able to reduce a large list that included species-specific changes into a much smaller list of core DEGs associated with stroma formation. Comparative analysis of these core-STRvSUB DEGs between sexual and asexual genomes further refined this list, identifying several interesting candidate genes implicated in having stroma-specific roles. This warrants further investigation into how these genes contribute to the virulent phase of the *Epichloë* life cycle. We were also able to leverage the SUB > STR expression of BGCs specifying host-protective natural products to identify 12 uncharacterized BGCs that may specify synthesis of novel host-protective natural products, along with 9 uncharacterized BGCs that exhibited STR > SUB expression, and 1 uncharacterized BGC that exhibited INF > SUB expression. The datasets we include provide a useful resource for future analyses, such as the investigation of conserved host gene expression changes associated with STR formation once genome sequences are available for these grass species.

## Data availability

Sequencing data generated for this project were submitted to NCBI with the draft genome for *Epichloë elymi* strain NFe728 available under Bioproject PRJNA623950, the RNAseq data from all SUB, STR, and INF samples available under Bioproject PRJNA554133, and the gene models generated for *E. elymi* NFe728, *E. festucae* E2368, and *E. typhina* E8 available under Bioproject PRJNA757131. Code generated for this project is available at https://github.com/klee8/stromata (last accessed 4 March 2022). The multiple sequence alignments used in the comparative genome analysis are included as supplementary files. Supplemental material is available at figshare: https://doi.org/10.25387/g3.16650658 (last accessed 4 March 2022).

## Funding

This research was supported by grants from the Royal Society of New Zealand Marsden Fund (MAU1502), the New Zealand Tertiary Education Commission through the Bio-Protection Research Centre, U.S. Department of Agriculture National Institute of Food and Agricultural Hatch project KY0102044 and Specific Cooperative Agreement 201809140939, and by Massey University.

## Conflicts of interest

None declared.
